# The motivation for citizens’ involvement in life sciences research is predicted by age and gender

**DOI:** 10.1371/journal.pone.0237140

**Published:** 2020-08-03

**Authors:** Martin Lakomý, Renata Hlavová, Hana Machackova, Gustav Bohlin, Maria Lindholm, Michela G. Bertero, Markus Dettenhofer

**Affiliations:** 1 Office for Population Studies, Faculty of Social Studies, Masaryk University, Brno, Czechia; 2 Faculty of Business and Economics, Mendel University in Brno, Brno, Czechia; 3 Institute for Research on Children, Youth and Families, Faculty of Social Studies, Masaryk University, Brno, Czechia; 4 Vetenskap & Allmänhet, Stockholm, Sweden; 5 Department of Learning, Informatics, Management and Ethics (LIME), Karolinska Institutet, Stockholm, Sweden; 6 Centre for Genomic Regulation (CRG), The Barcelona Institute of Science and Technology, Barcelona, Spain; 7 University Pompeu Fabra (UPF), Barcelona, Spain; 8 CEITEC – Central European Institute of Technology, Masaryk University, Brno, Czechia; Universitat Wien, AUSTRIA

## Abstract

Open Science is an umbrella term encompassing multiple concepts as open access to publications, open data, open education and citizen science that aim to make science more open and transparent. Citizen science, an important facet of Open Science, actively involves non-scientists in the research process, and can potentially be beneficial for multiple actors, such as scientists, citizens, policymakers and society in general. However, the reasons that motivate different segments of the public to participate in research are still understudied. Therefore, based on data gathered from a survey conducted in Czechia, Germany, Italy, Spain, Sweden, and the UK (*N* = 5,870), this study explores five types of incentives that can motivate individuals to become involved in life sciences research. The results demonstrate that men and younger individuals are more persuaded by extrinsic motives (external benefits or rewards), as compared with women and older people, who are driven by intrinsic motives (that originates from within an individual). This paper shows that specific strata of the population are differentially motivated to engage in research, thereby providing relevant knowledge for effectively designing public involvement activities that target various groups of the public in research projects.

## Introduction

Open Science strives to make scientific knowledge, including its processes and methods of production, more transparent, accessible, applicable, and responsive to the needs of researchers and society [1–3]. Open Science entails several approaches, such as *open data*, *open access to publications*, *open peer review* and *open source*, to cite a few, that aim to increase the accessibility, transparency and re-use of knowledge. Moreover, Open Science embraces the involvement of the general public and other stakeholders in the research process—so-called *citizen science* [1, 4]. Involving volunteers provides them with the opportunity to learn about research and its processes, to contribute directly to research projects with their intellectual capabilities and expertise, and to make a meaningful impact on science and society. Additionally, citizen science allows researchers to perform data collection and/or analysis on a much larger scale than otherwise possible, making science more effective in terms of time and financial costs [5].

According to the European Citizen Science Association, ‘Citizen science projects actively involve citizens in scientific endeavours that generate new knowledge or understanding’ [6]. Citizen science may denote the involvement of citizens at different stages of the research cycle, from initial discussions about various research topics and decision-making procedures to assistance with data collection and processing, and dissemination of knowledge [7, 8]. The benefits of citizen science include improvement of research quality for instance when volunteers help improve the research design [9], increased relevance of research to policymakers and laypeople, promotion of scientific knowledge and interest among different actors, increased agency provided to citizens, and promotion of dialogue between scientists and citizens [10–16]. When applied in specific contexts and disciplines, citizen science is likely to result in more desirable research findings [17–19]. However, only a minor proportion of the public has been involved in citizen science; the approximate number could be between two and three million participants worldwide according to the cited sources [20, 21].

There are several barriers to recruiting participants for citizen science projects, including the lack of motivation of potential participants themselves. In these projects, it is crucial to recognise the most effective ways to motivate different segments of the public to engage in the research process. This study intends to provide empirical evidence to help understand how people assess various incentives for public involvement. Following these goals, this study aims to answer the research question *What are the most effective incentives for participating in citizen science*?

## Theoretical background

### Motivating the public to engage in science

As motivation corresponds to an internal drive that is used to accomplish goals, incentive is, on the other hand, anything that can be used to motivate citizens towards that specific goal. Two distinct types of motivation [22] provide a way to explore different incentives for the public to engage in citizen science activities. First, intrinsic motivation originates from within an individual and indicates a person’s enjoyment of a certain activity [23]. Being intrinsically motivated to participate in research means, for example, being interested in the research topic, having the feeling to help society, or increase the impact of research. On the other hand, extrinsic motivation is connected to external benefits [24], such as rewards (e.g. money or awards), enhancement of reputation, self-image, or career opportunities. While the intrinsic motivation is crucial to various types of voluntary activities [25, 26], extrinsic motivation may be also important especially for attracting different segments of population [25].

Intrinsic motivation is shown to be more profound in volunteers in citizen science projects [27–30] in which members of the public participate in data collection and analysis development of research questions, methodology and communication of results. Tinati and colleagues [26] also show that it is not only difficult to recruit participants for a citizen science project, but also to keep them motivated and involved. Nevertheless, the above mentioned studies provide only limited evidence, since their findings are based upon a sample of citizen science participants who were mostly men with a higher level of education, and in addition had already been involved in other citizen science projects [27–29, 31]. Such limitations pertain to many studies of citizen science projects, which usually work with a small sample of volunteers with specific characteristics. Since intrinsic motivation is associated with more frequent prosocial behaviour [32] and altruism [33], we surmise, based on the findings of the abovementioned studies, that intrinsic motivation results in more lasting involvement than extrinsic motivation [34]. In contrast, external rewards seem to provide only short-term effects on involvement. When external incentives are no longer present, cooperation tends to decline over time [35]. The distinction between both types of motivation can deepen our understanding of different actors’ involvement in science.

### Differences in motivation based on individual characteristics

Intrinsic and extrinsic motivation have been shown to vary in relation to individual differences, such as gender, age, and education [36]. Inceoglu and colleagues [36] argue that people’s motives shift towards increased intrinsic motivation throughout a lifespan. As older people face many changes, such as declines in physical activity and information processing or more regular experiences of positive emotions, these changes may influence the salience of motives present in their lives that may affect overall motivation [36, 37]. Research suggests that intrinsic motives, such as autonomy, interest in the research topic, and values, gradually become more important in older age, and eventually replace extrinsic motives, which include competition, power, or material rewards [36, 38]. In line with Musick and Wilson [39], younger people are motivated to volunteer mainly by gaining experience and expanding their career possibilities in comparison to older people who are already in a job or retired. Older people tend to prefer activities leading to positive feelings for themselves, which is corroborated by Cappa and colleagues [5]. Similarly, competitiveness is replaced with cooperation in later life as social interactions help to obtain emotional satisfaction and support of one’s identity [38]. Some studies also observed the age-related differences in the involvement in citizen science. According to West and Pateman [40], participation in citizen science projects is the highest among middle-aged men, while Geoghegan et al. [29] report shows the highest participation in men aged 25–34 and 55–64 years.

Moreover, previous research suggests that the motives of participation may differ based on gender. Men are shown to value achievement and autonomy in the workplace [38, 41], whereas women tend to be more communal and altruistic [41, 42]. However, these tendencies become less salient with increasing age [36]. In academic settings, women are shown to have about the same levels of extrinsic motivation as men, but higher intrinsic motivation [43], with additional studies suggesting that female students have higher intrinsic, as well as extrinsic motivation [44]. Gender differences are also shown to be potentially explained by age, if both variables are present in the analysis, as illustrated by Inceoglu and colleagues [36]. In their study, older people were to a higher extent motivated by intrinsic motives than their younger counterparts, when controlled for gender and education. However, it is difficult to transpose any conclusions to involvement of participants in citizen science as the prior findings are rather inconsistent and drawn from different settings, including citizen science projects, workplace, and academia.

Finally, the level of education affects the willingness to be involved in several forms of scientific research of relevance for both simple and more complex tasks spanning from classifying or annotating graphical data, such as images of galaxies, to help formulating research questions [27, 45]. Regarding the intersection of gender and education, several studies have found a higher prevalence of highly educated men in citizen science projects [5, 27, 31]. The level of education is likely to be linked to specific motivations, since it is related to income, lifestyle, values, and other factors [46, 47]. One connection can be between the higher importance of material incentives and lower education [48], while others are hard to anticipate and can be culture-specific. Inceoglu and colleagues [36] found that the effects of education on certain types of motivation may be explained by the effect of age, but that it is important to control for the level of education in a multivariate analysis.

Interest in science and willingness to participate also need to be considered when exploring individuals’ motivation for involvement in science [49]. In line with this, prior research suggests that citizen science participants are, unsurprisingly, more likely to choose scientific topics that are interesting to themselves [27]. In conclusion, interest in science and willingness to participate need to be taken into consideration when exploring the motivation for participants in citizen science, as citizens who are more interested and willing to participate, may be more receptive toward a variety of incentives.

## Aims of the study

In this study, we aim to test how people evaluate the importance of five incentives for involvement in life sciences research and examine the differences in individual preferences among citizens. Considering the multiple beneficial outcomes of citizen science, it is crucial to understand which incentives for involvement are most effective. In prior research, several insights were gained, such as the variability in individual factors affecting overall motivation for participation [27, 36, 45]. However, much of the previous research was conducted on a self-selected group of citizen science participants, while this paper investigates potential participation in life-science research among representative samples of the public at large. To facilitate public involvement, we need better and more robust evidence concerning individuals’ motivation with further insight into inter-individual differences related to diverse motives. Such evidence would allow for more targeted and relevant ways to involve different subgroups in research, which, in turn, can positively influence citizens’ literacy and interest in science.

To fill these research gaps, our analysis is based on a survey involving representative samples from six European countries [50], assessing the influence that different individual characteristics have on incentives to trigger participation. In the survey, we analysed diverse incentives, including material rewards, public recognition, the involvement of personal acquaintances, help to society, and interest in the research topic. These incentives further segregate into intrinsic and extrinsic motivation [22–24]. Intrinsic motivation is exhibited by 1) help to society and 2) interest in the research topic; while extrinsic motivation is represented by 3) material incentives, 4) public recognition, and 5) involvement of personal acquaintances. To examine the individual differences in motivation, links to sociodemographic and background variables were included. Specifically, gender, age, education, interest in life sciences research, and willingness to participate were tested in relation to different types of incentives. Our study aims to disentangle their effect by controlling for their mutual effect. This is especially relevant for the control of education and country. The level of education is included in the analysis due to its connection to the effect of age. Similarly, the country of respondents needs to be controlled in the analysis, as we need to consider different cultural, institutional, and economic settings of six European countries, which may affect our findings. Thus, we included the variable “country” in the data analysis, but solely as a control variable, as it is beyond the scope of this paper to examine any cross-cultural variation of motivation for citizen science participation.

## Methods

### Procedure

This study utilises data from a public survey conducted as part of a European project, ORION (Open Responsible Research and Innovation to further Outstanding kNowledge). The project is funded by the European Commission under the Horizon 2020 framework programme [50]. The aim of the project is to embed the principles of Open Science in research funding and performing organizations with a specific focus on researchers, management staff and high-level leadership. The survey examined public attitudes towards involvement in science, and was conducted in Czechia, Germany, Italy, Spain, Sweden, and the UK, the six countries represented in the project. The data of the survey are freely accessible in the Zenodo repository under the name of the paper, in compliance with the Horizon 2020 Open Research Data pilot.

**Fig 1:**
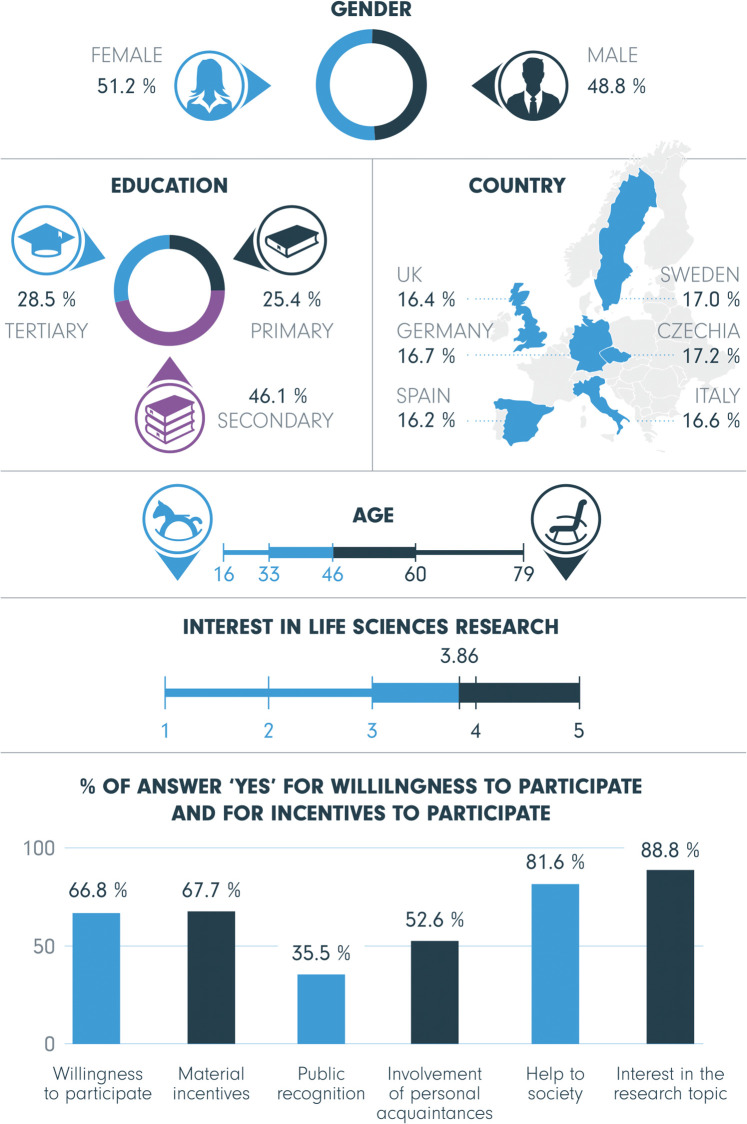
Descriptive statistics of the sample. The proportion of men and women (gender), education levels (primary, secondary, tertiary), countries of origin (UK, Germany, Spain, Sweden, Czechia, Italy) in our sample, and the distribution of participants’ age and interest in life sciences research (showing quartiles), the proportion of people who agreed they are willing to be involved in life sciences research personally and proportions of those motivated to be involved by five different incentives.

The data was collected by Nio Field Company using telephone interviews with approximately one thousand respondents in each country, which were chosen by Random Digit Dialling technique. Within each country, a stratified proportional sampling was used, accounting for age, gender, and education. The total nationally representative sample for the six countries comprises 5,870 respondents aged between 16 and 79. No personal identifying information is present in the data. The individual responses are linked only to categories of individual characteristics (country, age, sex, level of education), which are not possible to use for identification of a specific person. The response rate for each country was about 5%– 55% of the respondents were not available, 39% denied, and 1% had language problems—and thus suffers from a generally very low response rate of telephone interviewing via RDD [51, 52]. Respondents with missing values on some of the employed variables were excluded from the analysis, which yielded a final sample of 5,515 respondents. The basic characteristics of the sample are described in Fig 1. Two sets of binary logistic regression models [53] estimate which factors would increase the odds that the particular incentive would motivate respondents to get involved. The coefficients in the multivariate models show the effects of a variable while everything else is equal.

### Measures

#### The motivation for involvement in life sciences research

This paper examines the importance of different specific motivations across several demographic characteristics. Life sciences research was introduced by the sentence: ‘This research tries to understand living organisms such as human beings, animals, plants or bacteria and includes biology, genetics, neuroscience or medicine.’ The outcomes of the study measured the perceived role of five potential incentives for becoming involved in life sciences research. The following set of variables, indicating diverse incentives, represents the dependent variables in the analysis. The incentives were asked in the following way: ‘People can get involved in research for different reasons. What, if any, of the following would motivate you? The incentives included were *extrinsic*, specifically, a) Monetary or material incentives, b) Public recognition, c) If people I know were involved, and *intrinsic*, d) A belief that my involvement would help society, and e) If the research topic was interesting to me. The option No was coded 0 in each case.

#### Sociodemographic factors

The survey asked about age (range 16–79), gender (0 = female), level of education (1 = primary, 2 = secondary, and 3 = tertiary), and country of residence (Czechia, Germany, Italy, Spain, Sweden, and the UK).

#### Attitudes to life sciences research

The survey asked about overall interest in life sciences research and willingness to participate in life sciences research. *Interest in life sciences research* was measured by the question: ‘First, a question on how interested you are in life sciences research. Are you…’ with a Likert response scale ranging between very interested (= 5) and not at all interested (= 1). The final version of the scale was pre-tested in all languages in the same way as the rest of the questionnaire. The part of the questionnaire regarding *involvement in research* was introduced as follows: ‘People can receive information about research, but also contribute to research by sharing their own ideas, knowledge or experiences. They can participate by discussing research questions and methods, decide about funding or more directly by collecting, analysing or donating research material.’ *Willingness to participate in life sciences research* was measured by the question: ‘Would you consider being involved personally in life sciences research?’ with response option No coded 0. The analysis includes both respondents willing and not willing to participate at the time of the interview, as both of these groups still are valuable to consider for public involvement activities in research projects. The distribution of all variables in the sample is displayed in Fig 1.

## Results

### Descriptive results of the sample

Questions selected from the ORION public survey questionnaire for this study are presented in Table 1, in the same order as they were placed within the questionnaire. Descriptive statistics of the sample comprising 5,515 respondents are displayed in Fig 1. The sample consists of 2,692 (48.8%) men and 2,823 (51.2%) women, aged 16–79, with a mean age of 46 years (*SD* = 5.9). Close to half the sample (2,543; 46.1%) holds a secondary education degree, compared to 1,401 (25.4%) participants with primary education, and 1,571 (28.5%) with tertiary education. Participants are from six European countries, including Czechia, Germany, Italy, Spain, Sweden, and the United Kingdom (UK), represented by 16.2%–17.2% of the sample per country.

**Table 1:** A list of questions used in this paper presented in the same order as asked in the survey.

Variable	Question	Categories
**Age**	What is your age?	ages between 16 and 79
**Gender**	What is your gender?	male, female
**Education**	Which is your highest completed level of education?	country-specific levels coded into three categories: primary, secondary, tertiary
**Interest in life sciences research**	First a question on how interested you are in life sciences research. Are you …	very interested, fairly interested, neutral, not very interested, not at all interested
**Willingness to participate**	Would you consider being involved personally in life sciences research?	yes, no
**Incentives**	People can get involved in research for different reasons. What, if any, of the following would motivate you?	yes/no for each alternative: a) Monetary or material incentives b) Public recognition (e.g. if my name was mentioned in the project) c) If people I know were involved d) A belief that my involvement would help society e) If the research topic was interesting to me
**Country**	Collected automatically–respondents in each country interviewed in the official language of the country	Czechia, Germany, Italy, Spain, Sweden, the UK

‘Variable’ shows the names used in the analysis. ‘Question’ refers to the full questions asked in the survey. ‘Categories’ include the categories of answers derived from each Question.

### Effectiveness of incentives for involvement in life science research

Respondents were generally interested in life sciences research with a mean value of 3.86 on the scale from 5 (very interested) to 1 (not at all interested), and 66.8% expressed a willingness to participate in life sciences research. Regarding the incentives that would motivate respondents to be involved in life sciences research, the most effective were the two intrinsic incentives of *interest in the research topic* (88.8%) and *help to society* (81.6%). These were followed by extrinsic incentives: *material incentives* (67.7%), *involvement of personal acquaintances* (52.6%), and *public recognition* (35.5%).

### Binary logistic regression models for five different incentives

Table 2 presents models for two intrinsic incentives. We used the last categories of all categorical variables as reference categories. Higher age and being female predicted higher odds of being motivated by both these intrinsic incentives. Specifically, each year of age was associated with 1% higher odds for being motivated by both incentives, and women had 23–25% higher odds to be motivated than men. Furthermore, willingness to participate in life sciences research and higher interest in life sciences research predicted greater odds of being motivated by intrinsic incentives. In contrast, the effect of level of education was not significant. Finally, the respondent´s country was also included in the model, primarily to control for possible cross-country differences. We tested the effect of country by inclusion of a set of dummy variables with the UK as a reference category; that is, the effect of being from the UK was compared to being from each other country. Respondents from Czechia had higher odds of being motivated by both incentives (than those from the UK), and respondents from Spain had higher odds of being motivated by help to society. Other countries do not significantly differ from the UK.

**Table 2:** Binary logistic regression—Models with intrinsic incentives (help to society and interest in the research topics) as dependent variables.

	Help to society				Interest in the research topic			
	*B*	*S*.*E*.	*Sig*.	*OR*	*B*	*S*.*E*.	*Sig*.	*OR*
**Constant**	0.774	0.120	.000	**2.169**	1.400	0.143	.000	**4.054**
**Age**	0.011	0.002	.000	**1.011**	0.011	0.003	.000	**1.011**
**Gender–Male**	-0.263	0.075	.000	**0.769**	-0.290	0.091	.001	**0.749**
**Education**								
**Primary**	-0.045	0.108	.674	0.956	-0.250	0.128	.050	0.779
**Secondary**	0.046	0.093	.623	1.047	0.000	0.116	.998	1.000
**Tertiary (ref.)**								
**Interest in life sciences research**	-0.497	0.041	.000	**1.644**	-0.458	0.048	.000	**1.581**
**Willingness to participate**	1.157	0.086	.000	**3.181**	-1.290	0.104	.000	**3.632**
**Country**								
**Czechia**	1.088	0.135	.000	**2.969**	1.277	0.168	.000	**3.587**
**Germany**	-0.313	0.121	.010	**0.731**	0.020	0.147	.892	1.020
**Italy**	0.086	0.135	.528	1.089	0.122	0.164	.457	1.129
**Spain**	0.794	0.141	.000	**2.213**	0.337	0.155	.030	**1.401**
**Sweden**	0.130	0.122	.288	1.139	0.216	0.145	.137	1.241
**UK (ref.)**								

The table shows unstandardised (B) and standardised coefficients (odds ratios = OR; OR below 1 indicate negative effect and above 1 positive effect; OR = 1 indicates no effect), standard errors (S.E.), and statistical significance (Sig.). OR with p < .05 are in bold. Cox and Snell R^2^ and Nagelkerke R^2^ have values 0.12 and 0.19 for help to society and 0.08 and 0.17 for interest in the research topic.

Table 3 presents results from regression models for the three extrinsic incentives surveyed. In these models, opposite effects for age and gender in comparison to the models for intrinsic motivation have been shown. All of the extrinsic incentives had higher odds to motivate younger people, with each additional year of age lowering the odds of choosing the incentive by 2%, 4%, and 2%, respectively. Further, men had 20% higher odds to be motivated by material incentives and 45% higher odds to be motivated by public recognition, as compared to women. This means that women and older people had higher odds of being motivated by intrinsic incentives, while men and younger people had higher odds to be motivated by extrinsic incentives. There is no significant effect of level of education, but being interested in life sciences research and/or willing to get involved in life sciences research predicted higher odds of being motivated by all incentives. With regard to the country differences, as compared to the UK, people had higher odds of being motivated by: all three extrinsic incentives in Czechia, both material incentives and public recognition in Germany and Spain, and by public recognition in Italy.

**Table 3:** Binary logistic regression—Models with extrinsic incentives (material incentives, public recognition, and involvement of personal acquaintances) as dependent variables.

	Material incentives				Public recognition				Involvement of personal acquaintances			
	*B*	*S*.*E*.	*Sig*.	*OR*	*B*	*S*.*E*.	*Sig*.	*OR*	*B*	*S*.*E*.	*Sig*.	*OR*
**Constant**	-0.192	0.101	.056	0.826	-1.678	0.110	.000	**0.187**	-0.389	0.095	.000	**0.677**
**Age**	-0.018	0.002	.000	**0.982**	-0.036	0.002	.000	**0.964**	-0.016	0.002	.000	**0.984**
**Gender–Male**	0.183	0.061	.003	**1.201**	0.370	0.061	.000	**1.448**	0.109	0.056	.050	1.116
**Education**												
**Primary**	-0.121	0.087	.164	0.886	0.133	0.086	.123	1.142	0.069	0.080	0.386	1.071
**Secondary**	0.011	0.075	.886	1.011	0.100	0.075	.185	1.105	0.013	0.068	0.854	1.013
**Tertiary (ref.)**												
**Interest in life sciences research**	0.133	0.035	.000	**1.142**	0.293	0.038	.000	**1.341**	0.208	0.033	.000	**1.231**
**Willingness to participate**	0.880	0.073	.000	**2.412**	0.580	0.077	.000	**1.786**	0.410	0.068	.000	**1.507**
**Country**												
**Czechia**	1.127	0.112	.000	**3.086**	0.538	0.111	.000	**1.712**	0.655	0.101	.000	**1.925**
**Germany**	0.275	0.104	.008	**1.316**	0.337	0.109	.002	**1.401**	0.245	0.098	.013	**1.277**
**Italy**	0.142	0.107	.184	1.152	0.524	0.108	.000	**1.690**	-0.059	0.100	.557	0.943
**Spain**	0.579	0.108	.000	**1.785**	0.472	0.107	.000	**1.603**	-0.115	0.098	.240	0.892
**Sweden**	-0.039	0.100	.695	0.961	0.129	0.109	.236	1.138	0.131	0.096	.174	1.140
**UK (ref.)**												

The table shows unstandardised (B) and standardised coefficients (odds ratios = OR; OR below 1 indicate negative effect and above 1 positive effect; OR = 1 indicates no effect), standard errors (S.E.), and statistical significance (Sig.). OR with p < .05 are in bold. Cox and Snell R^2^ and Nagelkerke R^2^ have values 0.09 and 0.12 for material incentives, 0.12 and 0.17 for public recognition, and 0.05 and 0.07 for involvement of personal acquaintances.

A possible moderating effect of each predictor (age, gender, interest in life sciences research) was tested via inclusion of interactions between predictors for each regression model from Tables 2 and 3, but none of them improved the models; thus, based on our data, we cannot conclude that there is any of the tested moderation effects. Finally, the values of R^2^ ranging from 0.05 to 0.19 indicate weak to moderate explanatory power of the models.

### Predicted probabilities for individual characteristics

**Fig 2:**
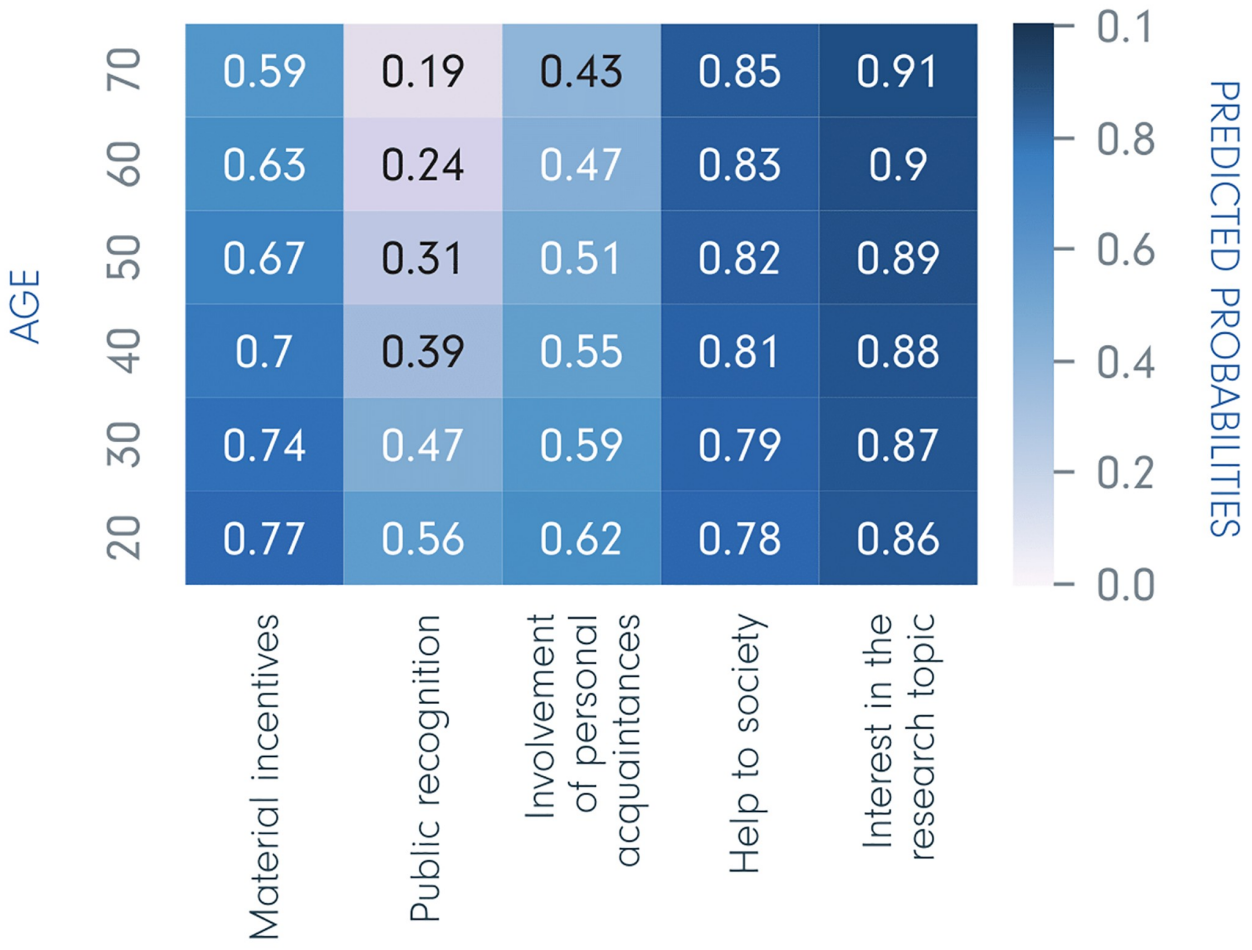
Age—Predicted probabilities of being motivated by the five incentives.

Based on the logistic regression models, we estimated the predicted probabilities (0 to 1) of being motivated by each of the five incentives, depending upon individual demographic characteristics. Fig 2 shows predicted probabilities for respondents of different ages displayed in the form of a heatmap. This figure shows the probability of selecting a given incentive predicted by the regression model for a person of a certain age; for instance, 20-year-olds have a probability of 0.56 for selecting public recognition when other characteristics are controlled for. Hence, the colours show that, a) each of the incentives are of different importance, i.e. placed at different parts of the scale 0–1, and b) the probability of extrinsic motivation decreases with age, while the probability of intrinsic motivation increases with age. Moreover, all these associations among incentives and age are significant (*p* < .001). The largest effect of age is found for public recognition, with a probability of 0.56 for 20-year-olds, 0.31 for 50-year-olds, and 0.19 for 70-year-olds. In contrast, respondents aged 20 years have 0.78 predicted probability of choosing help to society, while respondents aged 70 have 0.85.

**Fig 3:**
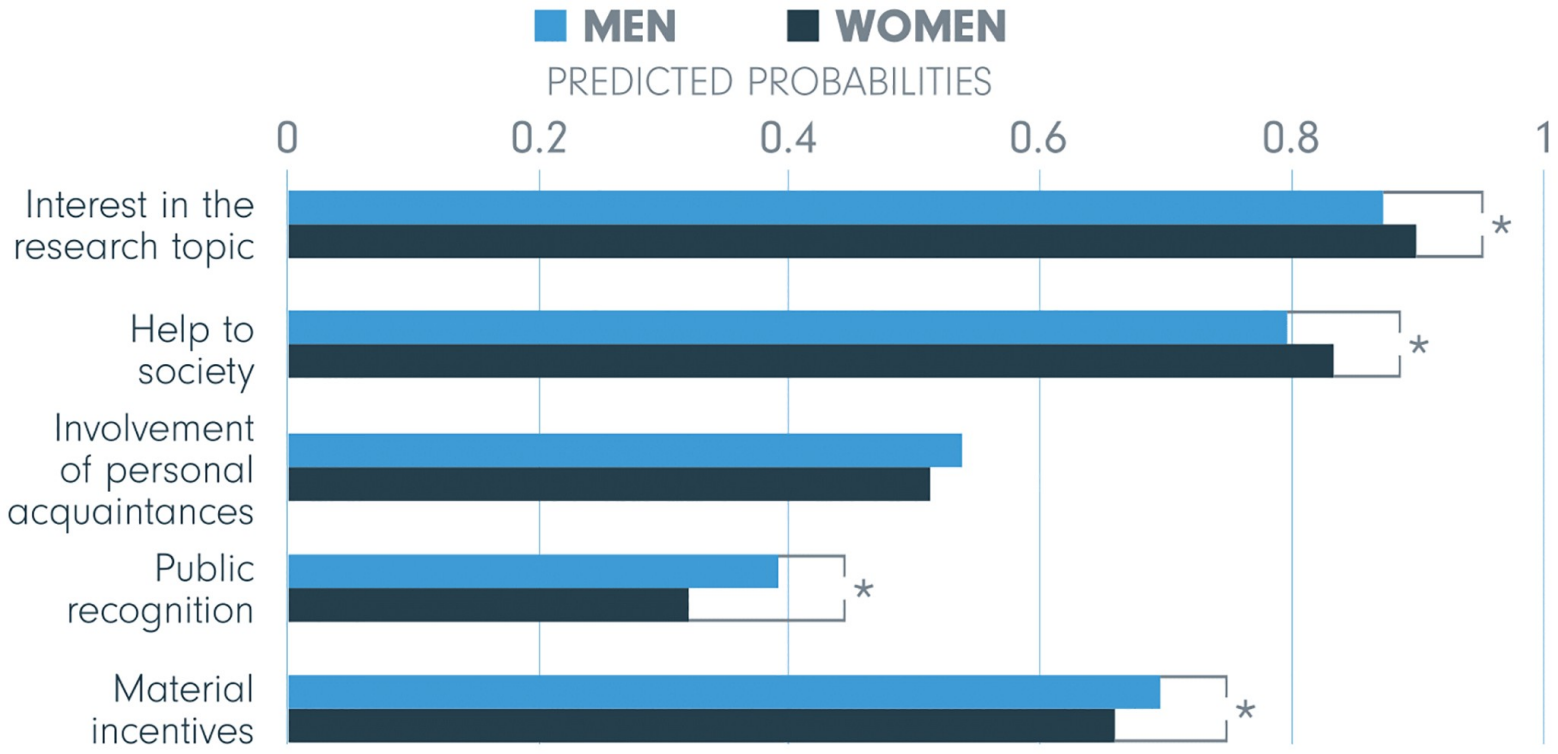
Gender—Predicted probabilities of being motivated by the five incentives. The significant differences are indicated by an asterisk (*) indicating *p* < .05.

The difference between men and women was significant for four out of five incentives (indicated by an asterisk, *), and predicted probabilities displayed in Fig 3 show how the effect of gender differs for extrinsic and intrinsic motivation. On the one hand, two out of three extrinsic incentives are more important to men—material incentives with predicted probabilities 0.70 and 0.66 and public recognition with 0.39 and 0.32. On the other hand, predicted probabilities for both intrinsic incentives are significantly higher for women (0.83 versus 0.80 for help to society and 0.90 to 0.87 for interest in the research topic). Finally, no significant difference is found for involvement of personal acquaintances as a motive for involvement.

**Fig 4:**
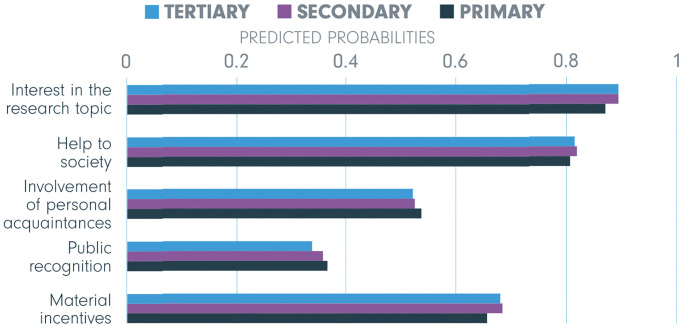
Educational background—Predicted probabilities of being motivated by the five incentives. Significant differences (*p* < .05) would be indicated by an asterisk (*), but no significant differences were found amongst educational groups.

**Fig 5:**
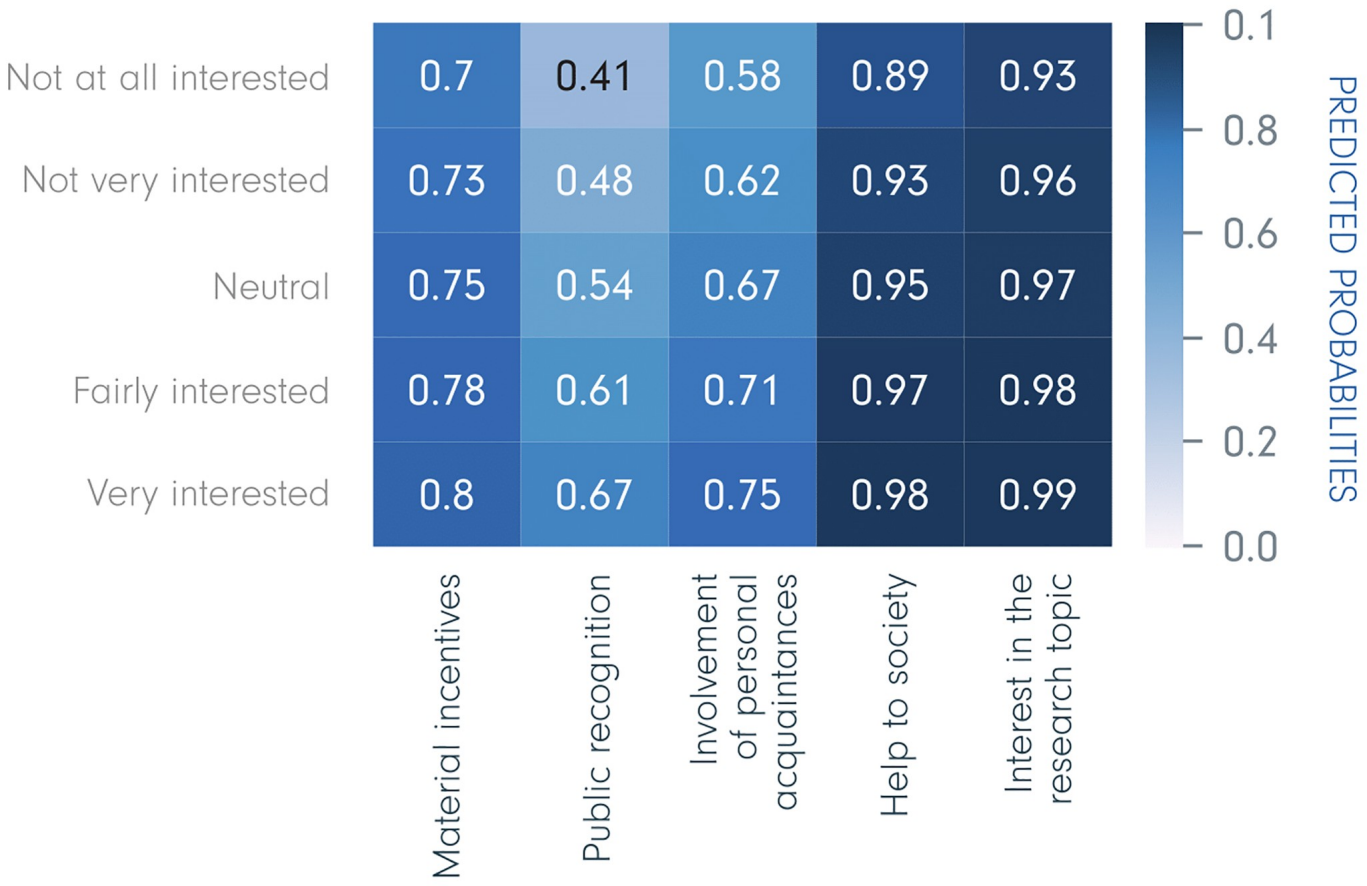
Interest in life sciences research—Predicted probabilities of being motivated by the five incentives.

**Fig 6:**
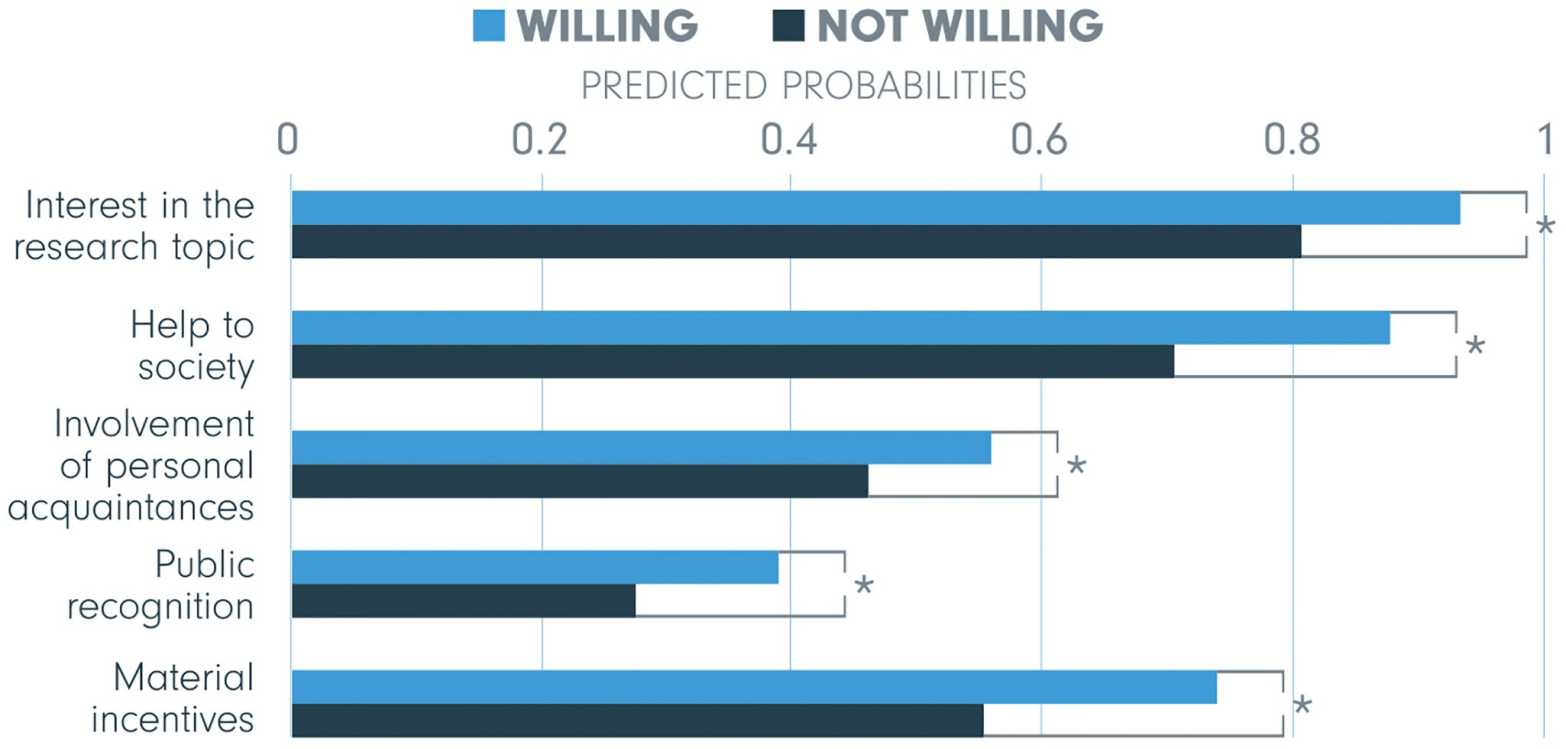
Willingness to participate in life sciences research—Predicted probabilities of five incentives. Significant differences (*p* < .05) are indicated by an asterisk (*).

Figs 4–6 display predicted probabilities for other individual characteristics. Fig 4 shows that differences in incentives based on the level of education are negligible (and the effects were not significant). In contrast, both interest in life sciences research and willingness to participate in life sciences research significantly predicted odds of being motivated by the incentives. Differences in probabilities between respondents who are very vs. not at all interested in life sciences research vary between 0.06 and 0.26 across all incentives (differences between the top and the bottom row in Fig 5). Differences between those who are willing and those who are not willing to be involved are presented in Fig 6 and vary between 0.10 and 0.19 across all indicated incentives. Finally, while there was a clear and opposite pattern in associations of extrinsic vs. intrinsic motivation with age and gender. Other predictors show differences only in magnitude but not in the direction of the links.

## Discussion

This paper examines ways to motivate citizens to be involved in life sciences research, with a specific focus on the role of individual characteristics. Data from a public survey conducted on representative samples from six European countries—Czechia, Germany, Italy, Spain, Sweden, and the UK—were used to address this topic. The findings shed some additional light on how to motivate citizens to engage in citizen science. Insights into the effectiveness of different incentives for subgroups of the general public can generate a thorough reflection on how to engage a wider and more diverse group of citizens than those who would already be involved in such projects.

Generally, citizens indicated that their interest in the research topic would strongly motivate them to participate, as the other intrinsic motivation—the prospect that their involvement could help society. In contrast, material incentives, the involvement of personal acquaintances, and public recognition (ordered from the most to the least important factors), reflecting extrinsic motivation, were less important. This is in line with West and Pateman [40] who identified intrinsic motivation as the most important for participating in citizen science projects, including the desire to help and to contribute to scientific knowledge, while the desire to please others was less important. Similarly, Domroese and Johnson [54] suggest that interest in the topic and contributing to science are the most important motives for participating in citizen science projects.

Our examination uncovered differences based on individual characteristics. These differences were in line with our expectations, based on the findings from prior studies. First, our findings suggest that extrinsic motivation is more important for men than women, while intrinsic motivation is more important for women than men. Second, extrinsic motivation was more important for younger respondents, while intrinsic motivation was more important for older respondents, which is in line with previous research from the area of work motivation [36, 38], as well as volunteering, including Musick and Wilson [39], for example. Third, we did not find any link to the level of education. This result corroborates the findings of Inceoglu and colleagues [36], who argue that the effects of education may be potentially explained by age, if both variables are included in the analysis. Fourth, higher interest in life sciences research and willingness to participate were positively associated with all types of incentives. Finally, to account for possible country differences in our examination, we included also the respondent’s country in our models. There were some country differences in the estimated effect of diverse incentives; however, deeper interpretation of these findings is beyond the scope of the study presented here.

While previous research has been conducted on self-selected groups of citizen science participants, this paper focuses on the willingness of the citizens to participate in research in life sciences, in general. This factor can be considered both a limitation, given the general topic and no direct connection to actual behaviour, and a strength of the presented study, since it provides information from a representative sample of respondents. Our findings show that both intrinsic and extrinsic motivation have relevance when targeting individuals’ potential involvement in science, and citizen science, specifically. As we gain more knowledge of why people participate in science, the projects can be better targeted to attract more citizens. Our study indicates that, generally, intrinsic motivation is most important, especially for women and older people, when it comes to motivating the public to be involved in life sciences research. Extrinsic motivation may, in contrast, prove more helpful when targeting men and younger people.

The study has two main limitations that are common for work with questionnaire-based data. First, the data are based on self-reported statements and not on records of actual behaviour. Some respondents might have over- or underestimated the importance of the incentives [55]. We also expect a certain overestimation of the willingness to become involved, as the respondents already volunteered to answer the phone survey. Moreover, the answers may be influenced by a special type of response error called social desirability, which stems from the human tendency to present themselves in a way appreciated by other members of society and is especially relevant for some of the survey items [56]. Further, a proclaimed willingness to do something via telephone might differ from the willingness in a real-life situation [57, 58]. This limitation as a whole implies that the data may not be fully congruent with actual behaviour. Still, this topic is hard to study in experimental settings, although, the selected methodology is a standard procedure within social sciences. Second, the questionnaire asked about five incentives chosen by the team of experts from the ORION project, while some additional incentives may be important for motivating participants. This second possible limitation can be addressed only by additional research on the topic.

Despite these limitations, this paper brings relevant data and conclusions to help reflecting on strategic decisions for engaging participants from different population groups. Most citizen science projects with basic requirements for expertise tend to attract certain population groups to a higher extent, for example, middle-aged male with higher education [27, 45]. Our findings provide guidance for designing more effective ways of recruiting participants with regard to key individual characteristics. It also sheds more light on the barriers in participation related to specific incentives (e.g., low interest in life science research) within the general public. The future research should be built upon the main weakness of similar studies—questionnaire-based data—and expand the knowledge by experimental or unobtrusive research.
